# Improving diagnosis of inherited peripheral neuropathies through gene panel analysis

**DOI:** 10.1186/s13023-016-0500-5

**Published:** 2016-08-22

**Authors:** Petra Laššuthová, Dana Šafka Brožková, Marcela Krůtová, Jana Neupauerová, Jana Haberlová, Radim Mazanec, Pavel Dřímal, Pavel Seeman

**Affiliations:** 1 Department of Paediatric Neurology, DNA Laboratory, 2nd Faculty of Medicine, Charles University in Prague and University Hospital Motol, Prague, Czech Republic; 2 Department of Paediatric Neurology, 2nd Faculty of Medicine, Charles University in Prague and University Hospital Motol, Prague, Czech Republic; 3 Department of Neurology, 2nd Faculty of Medicine, Charles University in Prague and University Hospital Motol, Prague, Czech Republic

**Keywords:** Inherited peripheral neuropathies, Charcot-Marie-Tooth, Targeted gene panel testing, Mutation, Phenotype

## Abstract

**Background:**

Inherited peripheral neuropathies (IPN) are the most common inherited neurological condition. It represents a highly heterogeneous group, both clinically and genetically.

Targeted disease specific gene panel massively parallel sequencing (MPS) seems to be a useful tool in diagnosis of disorders with high genetic heterogeneity.

**Methods:**

In our study, we have designed, validated and updated our own custom gene panel of all known genes associated with IPN. One hundred and ninety-eight patients have been tested so far. Only patients in whom mutations in more common causes or relevant genes have already been excluded were enrolled. Five consecutive panel designs were prepared according to recent literature search, the last one covering ninety-three genes. Each patient was tested only once. All data were evaluated with at least two different pipelines.

**Results:**

In summary, causative mutation has been found in fifty-one patients (26 %). The results were inconclusive in thirty-one (16 %) patients. No variants of likely significance to IPN were found in one hundred and sixteen (58 %) patients.

**Conclusion:**

MPS gene panel enables testing of all known IPN causes at once with high coverage and at an affordable cost making it truly a method of choice also in IPN. Gene panel testing results in several interesting results and findings.

**Electronic supplementary material:**

The online version of this article (doi:10.1186/s13023-016-0500-5) contains supplementary material, which is available to authorized users.

## Background

Inherited peripheral neuropathies (IPN) are the most common inherited neurological condition, with a reported prevalence 1/2500 [1]; a prevalence 1/1214 has also been noted [2]. IPN is an example of a genetically highly heterogeneous group of disorders. Mutations in more than 90 genes are already associated with IPN [3, 4].

Our Center for inherited neuropathies in Charles University in Prague and University Hospital Motol is unique for the whole Czech Republic. In the course of 17 years, we have collected DNA samples and clinical data from more than 3100 patients from 2155 independent families. The cause of inherited neuropathy has been stated in 920 unrelated families (1775 patients), so far.

In the previously diagnosed 920 families, *PMP22* duplication was detected in 412 families (772 patients), *PMP22* deletions were detected in 290 families (485 patients) and in the remaining 218 families (518 patients) causative point mutations in known CMT genes were detected.

However, molecular genetic diagnosis was still unknown in approximately 1378 patients (from 984 unrelated families). Sanger sequencing of individual genes is time consuming and not very successful for many less frequent causes of IPN. Targeted resequencing with a gene panel was therefore a promising option in such situations. It offers the possibility of sequencing all genes, mutations of which are associated with inherited neuropathies, in real time, in massively parallel mode.

## Methods

The study was approved by the ethics committee of University Hospital Motol and informed consent was obtained from all patients. A permission was obtained to publish the personal information essential for the understanding of the manuscript.

### Patients

One hundred and ninety-eight patients (affected unrelated patients) from 198 unrelated families were included in the study. These patients were selected according to the following criteria:

1. IPN phenotype (peripheral neuropathy motor and/or sensory) supported with nerve conduction studies, with no other detectable acquired cause;
2. Availability of other family members for molecular- genetic testing;
3. Patients were previously tested for CMT1A duplications and HNPP deletions in relevant cases. Moreover, most relevant or common IPN genes have already been tested in all patients with Sanger sequencing dependent on the provided clinical and electrophysiological and family data and these tests did not identify causal mutation; this is shown in Additional file 1: Part 1/figure A.

From the patients included in the study: fifty-nine patients were reffered with demyelinating neuropathy (HMSN I), ninety-three patients were reffered with axonal neuropathy (HMSN II). Eight patients were classified as having intermediate neuropathy. The remaining patients were classified as having HMSN or IPN without more details (Additional file 1: Part 1). The majority of selected patients were sporadic cases (Additional file 1: Part 1).

**Fig. 1:**
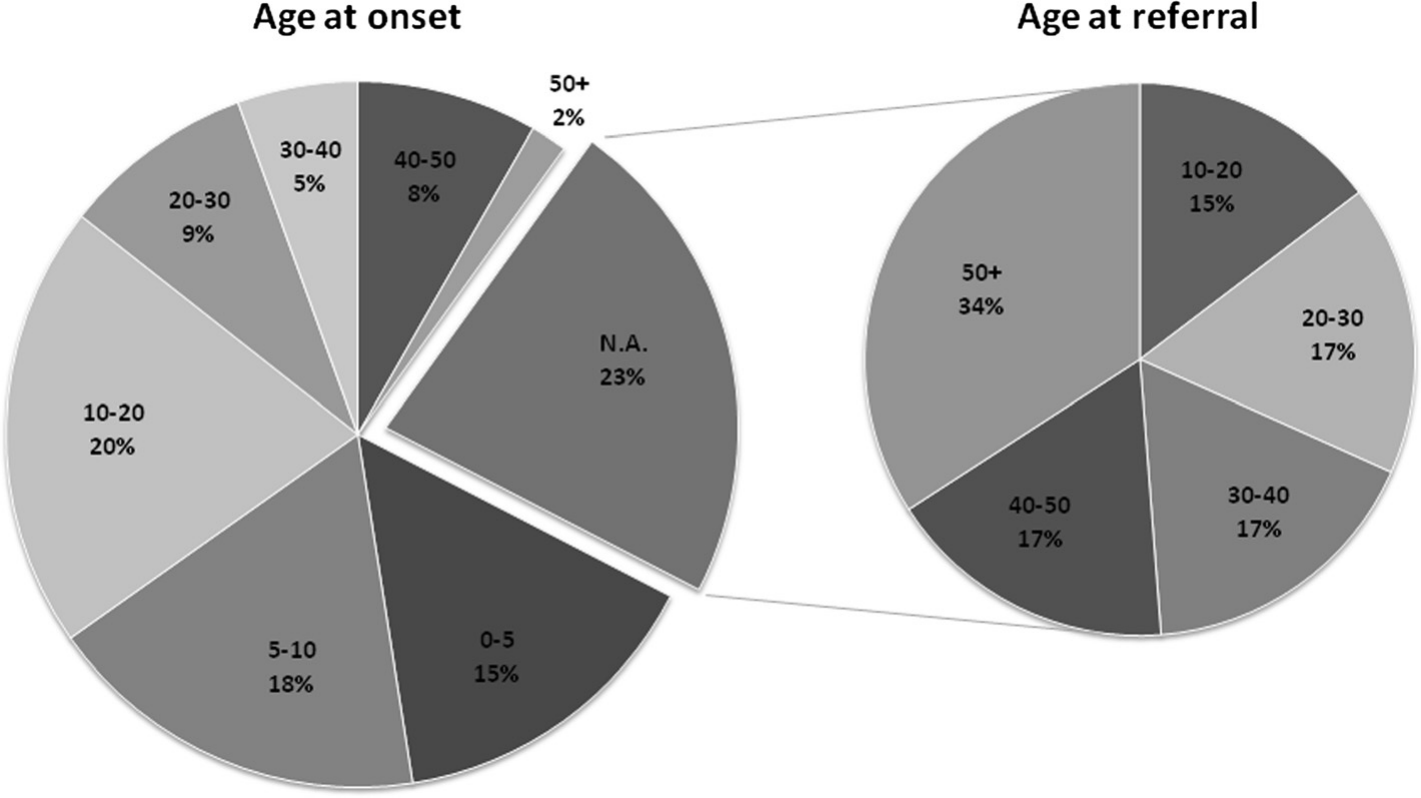
Age at onset of the disease (study cohort). For 77 % of probands included in the study the data about the age at onset of the disease were available. These are represented in the graph Age at Onset. For 23 % of probands these data were not readily available (N.A./ not available), however, age at referral for these probands is shown, as this may also be a useful surrogate (graph Age at referral). Each block in the graph represents a different age group; age groups are described as intervals in years, and percentage of probands with onset/referral is shown. From probands included in the study, 60 % of them had onset of the disease before the age of 20 years

Patients were referred to our department by neurologists, clinical geneticists and neuromuscular centres from the whole Czech Republic over a period of 17 years (1998–2015). The age of onset of the disease in probands tested in this study is shown in Fig. 1. Sixty percent of patients had the age of onset before the age of 20 years.

### Targeted resequencing

HaloPlex technology (Agilent Technologies, Santa Clara, USA) was used.

### Design

NGS HaloPlex target enrichment library design was created with SureDesign application provided by Agilent (Agilent Technologies, Santa Clara, CA, USA). Genes were included in the design based on these criteria:

1. At least two independent literature reports exist (“**known IPN genes**”).
2. **“New” genes** were also included in the design, if the primary report presented:
  - evidence for pathogenic mutations in at least two unrelated families
  - evidence for pathogenic mutation in only one family; The family was larger than a nuclear family and evidence was supported by functional studies.
3. Also, **five candidate IPN genes** were included in the design. The idea behind this was to search for a second family with mutation in the same gene and similar IPN phenotype, and thus to”confirm” the gene.

Three genes were selected from a paper by Schabhuttl et al. [5]: *SH3PB4, ITPR3* and *KLHL13* genes.

Moreover, *SLC18A3* gene was also included in the design, based on our WES study in one Czech family. A possibly pathogenic de-novo mutation has been detected with trio analysis in this gene by the de-novo approach. However, the phenotype in this family is very unique [6] and we have not yet been able to find the second family.

Also, mutation in *MICAL1* gene has been found in one Czech family with combination of linkage and WES analysis. However, further studies and analyses have shown that the variant is probably only a VUS (variant of unknown significance), and the relationship between *MICAL1* gene and IPN has not been proved. This gene will therefore not be included in future designs.

Overall, five consecutive designs were prepared. A list of genes is available in Additional file 1: Part 2.

### Sequencing

Prepared libraries were pooled and sent out for sequencing, performed in an outsorced laboratory – EMBL genomics core facilities (Heidelberg, Germany). Instrument MiSeq from Illumina was used (Illumina, Inc., San Diego, CA, USA).

### Analysis

#### SureCall analysis

##### SureCall default

Firstly, all fastq files were analyzed in SureCall application with default settings (Agilent_SureCall_2.1.0.21). Afterwards, for every sample a vcf file was generated with no filters applied. These vcf files were then merged into one vcf file with Galaxy installation of the vcf combine tool [7]. Merged vcf was then evaluated in several steps. In the first step, this vcf file was loaded into GenomeTrax tool (BioBase International, Qiagen Company, MA, USA). This tool identified all known mutations, previously reported in Human Gene Mutation Database (HGMD® Professional, BioBase International, Qiagen Company, MA, USA). Merged vcf was also annotated with Annovar [8].

##### SureCall coverage10

Secondly, files were analysed in the SureCall application with more relaxed settings. This analysis method has been called SureCall coverage10 for working purposes. Differences from default settings are mainly in SNP filter settings. The aim of this analysis was to call variants that were covered sufficiently, but not called – because the default settings in SureCall are set to call variants covered at least 40x. Analysis SureCall coverage10 was set to call variants covered at least 10x. This has raised the false positives, however, we decided to include this step in analysis because:

1. This approach has led to finding the causal mutation in five more patients. These mutations would have been missed by default approach only.
2. This approach was used as a second step, and a combination of default settings analysis in the first step with relaxed analysis in the second step has turned out to be most effective as this is combination of high specificity (default settings) and high sensitivity (relaxed settings) analysis process.

Vcf files were generated in the same manner, and analysed similarly as reported above.

#### Galaxy analysis

For high sensitivity of the analysis, we also decided to include in the workflow an analysis with our own pipeline based in Galaxy[7]. The schematic representation of the pipeline and Galaxy pipeline parameters and pipeline resources are listed in Additional file 1: Part 3.

#### In-house database of variants in MySQL

All three analyses were processed for all samples. For our own purposes and for comprehensibility we developed our own in-house database of variants in the MySQL environment.

#### Variants evaluation

All called variants were subjected to a review.

Variants were classified as:

1. rare benign variants if having EVS frequency more than 4 % or were present in more than 3 samples out of 48 or more (one run) or cumulative frequency was more than 5 alleles out of 198 patients.
2. remaing variants were evaluated with Alamut software (Interactive Biosoftware, http://www.interactive-biosoftware.com/). Called variants were classified according to recommendation by American College of Medical Genetics and Genomics (ACMG) [9].

#### Variants confirmation

Variants listed as DM and DM? in HGMD® Professional OR classified as pathogenic or likely pathogenic (category 1. or 2. according to ACMG) OR which fulfilled criteria in Additional file 1: Part 4 were further analyzed and were considered to be **variants of interest**.

Firstly, **variants of interest** were confirmed by Sanger sequencing. During the process a relatively large number of primers for PCR must be designed. We have used Optimus primer [10]. Afterwards, if a variant has been confirmed with Sanger sequencing, we proceeded to segregation analysis of the variant with the disease in the family. All other available family members were tested for presence or absence of this variant.

Importantly, the phenotype of the patient was evaluated in the scope of the tested variant.

#### Drawing a conclusion about a variant

Based on a recommendation from ACMG [9] the following conclusions have been made:

1. “positive”: A detection of mutation that explains a patient’s condition.
  For variants previously reported as pathogenic, confirmed by Sanger sequencing and also segregation of the variant with the disease in the family, was tested and proved. Moreover, the clinical phenotype of the patient was critically evaluated against the literature reports and our own clinical experience.
  This conclusion was made also for novel variants in known IPN genes, if the variant fullfiled criteria in Additional file 1: Part 4 and again the variant was confirmed by Sanger sequencing and also segregation of the variant with the disease in the family, and was tested and proved. Also, the phenotype of the patient is consistent with what has been described before in the literature and our own clinical experience.
2. “negative” (no variants identified of likely relevance to the diagnostic indication)
  For this conclusion to be made, we required the NGS data to pass quality criterion: at least 97 % of targeted bases is covered at least 10x. Otherwise, the library for the sample would have been prepared again.
3. “inconclusive” (a clear explanation of the patient’s condition was not found either due to only variants of unknown significance being identified or due to only a single heterozygous variant identified for a recessive condition)”

### CNV analysis

Data for the remaining one hundred and forty-seven patients were further tested with other algorithms with the aim of finding the causal mutations. Copy number variation analysis was performed with three different approaches – firstly, SureCall analysis (Agilent Technologies, CA, USA) predicts CNVs based on log ratio of the normalized sample read depth to the reference sample read depth. Secondly, NextGene (SoftGenetics LLC., PA, USA) analysis based on hidden Markov model (HMM) and lastly, NextGene analysis based on data normalization (in beta testing version) were performed.

## Results

### Causal variants

**Fig. 2:**
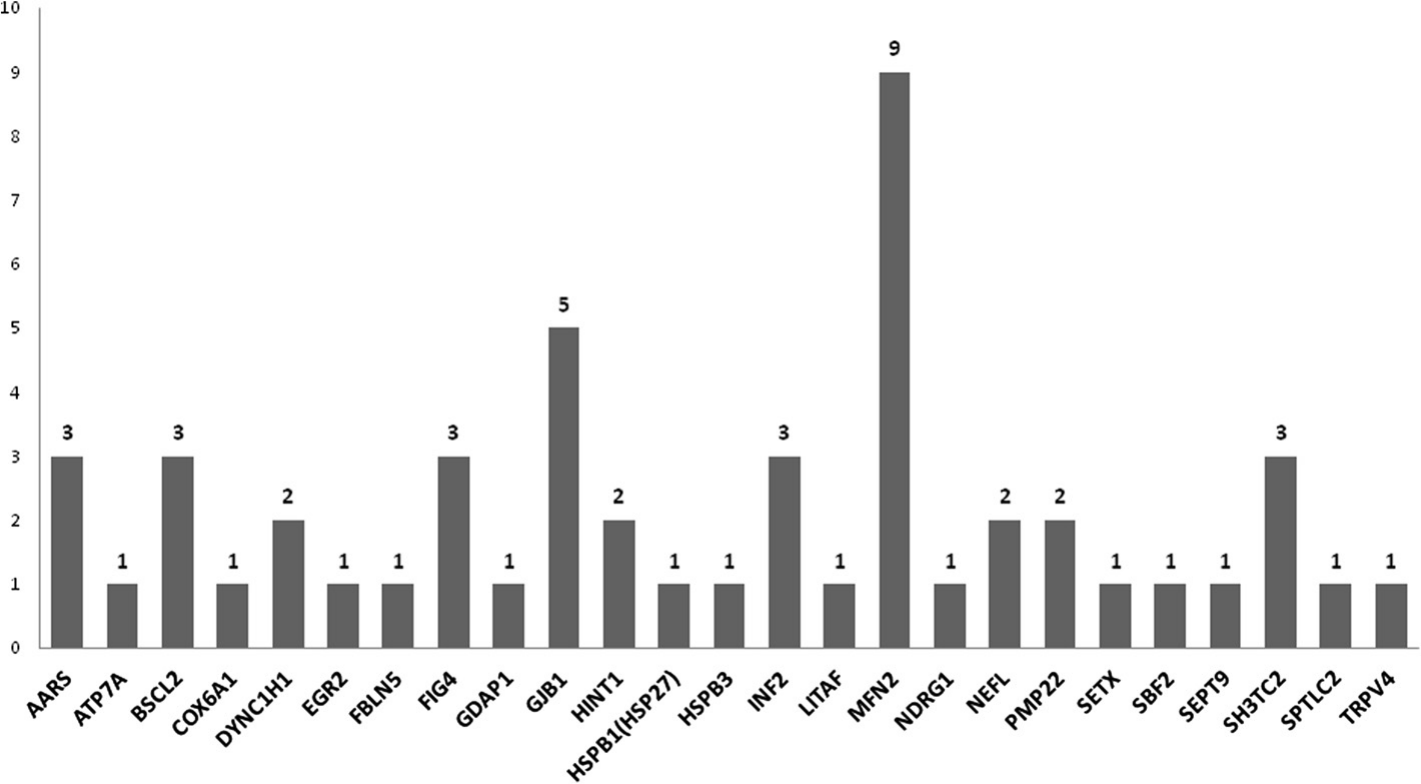
Causal variants in these genes have been found. Legend: X – axis: genes; Y – axis: number of patients with causal mutation

Causative mutations have been found in 51 independent patients (Fig. 2) and in 21 genes. Pathogenic mutations detected in the study and genotypes of the patients in whom the genetic diagnosis was established are presented in Table 1.

**Table 1:** Genotypes of patients with pathogenic mutations

Gene	Ref. sequence	No. of unrelated families with the mutation	Variations at DNA-level (relative to coding DNA sequence)	Variation at protein level (deduced)
*AARS*	(NM_001605.2)	3	c.986G>A	p.Arg329His
*ATP7A*	(NM_000052.6)	1	c.2981C>T	p.Thr994Ile
*BSCL2*	(NM_032667.6)	2	c.263 A>G	p.Asn88Ser
*BSCL2*	(NM_032667.6)	1	c.269 C>T	p.Leu90Ser
*COX6A1*	(NM_004373.2)	1	c.[247-7_247-3del];[c.247-7_247-3del]	
*DYNC1H1*	(NM_001376.4)	2	c.1792 C>T	p.Arg598Cys
*EGR2*	(NM_000399.3)	1	c.1231G>A	p.Asp411Asn
*FBLN5*	(NM_006329.3)	1	c.1117C>T	p.Arg373Cys
*FIG4*	(NM_014845.5)	1	c.[498-1G>A];[122T>C]	
*FIG4*	(NM_014845.5)	2	c.[793C>T];[122T>C]	p.[Arg265*];[Ile41Thr]
*GDAP1*	(NM_018972.2)	1	c.715C>T	p.Leu239Phe
*GJB1*	(NM_000166.5)	1	c.88A>T	p.Ile30Phe
*GJB1*	(NM_000166.5)	1	c.641dup	p.Arg215Profs*28
*GJB1*	(NM_000166.5)	1	c.622G>A	p.Glu208Lys
*GJB1*	(NM_000166.5)	1	c.212T>C	p.Ile71Thr
*GJB1*	(NM_000166.5)	1	no amplification (deletion), confirmed with MLPA	
*HINT1*	(NM_005340.5)	2	c.[110G>C];[110G>C]	p.[Arg37Pro];[Arg37Pro]
*HSPB1(HSP27)*	(NM_001540.3)	1	c.505_506dup	p.Met169Ilefs*5
*HSPB3*	(NM_006308.2)	1	c.21G>T	p.Arg7Ser
*INF2*	(NM_022489.3)	1	c.383T>C	p.Leu128Pro
*INF2*	(NM_022489.3)	1	c.233T>C	p.Leu78Pro
*INF2*	(NM_022489.3)	1	c.162-173del	p.Lys55_Glu58del
*LITAF*	(NM_001136472.1)	1	c.334G>A	p.Gly112Ser
*MFN2*	(NM_014874.3)	1	c.280 C>T	p.Arg94Trp
*MFN2*	(NM_014874.3)	1	c.436 C>T	p.Leu146Phe
*MFN2*	(NM_014874.3)	1	c.493 C>T	p.His165Tyr
*MFN2*	(NM_014874.3)	1	c.701T>A	p.Met234Lys
*MFN2*	(NM_014874.3)	1	c.839G>A	p.Arg280His
*MFN2*	(NM_014874.3)	1	c.880C>T	p.Arg294*
*MFN2*	(NM_014874.3)	1	c.1081C>T	p.His361Tyr
*MFN2*	(NM_014874.3)	1	c.1090 C>T	p.Arg364Trp
*MFN2*	(NM_014874.3)	1	c.1574A>G	p.Asn525Ser
*NDRG1*	(NM_001135242.1)	1	c.442 C>T	p.Arg148*
*NEFL*	(NM_006158.4)	1	c.310T>G	p.Phe104Val
*NEFL*	(NM_006158.4)	1	c.1186G>A	p.Glu396Lys
*PMP22*	(NM_153322.1)	1	c.124T>C	p.Cys42Arg
*PMP22*	(NM_153322.1)	1	c.421_436del	p.Val141Profs*9
*SETX*	(NM_015046.5)	1	c.[1656G>T(;)1658C>T]	p.[(Gln552His(;)Ser553Phe)]
*SBF2*	(NM_030962.3)	1	c.[134T>A];[c.134T>A]	p..[Ile45Asn];[Ile45Asn]
*SEPT9*	(NM_001113491.1)	1	heterozygous large duplication, confirmed with MLPA	
*SH3TC2*	(NM_024577.3)	1	c.[2860C>T];[c.279G>A]	p.[Arg954*];[Lys93Lys]
*SH3TC2*	(NM_024577.3)	1	c.[2860C>T];[1447T>G]	p.[Arg954*];[Phe483Val]
*SH3TC2*	(NM_024577.3)	1	c.[2860C>T];[c.2812C>T]	p.[Arg954*];[His938Tyr]
*SPTLC2*	(NM_004863.3)	1	c.1144G>C	p.Gly382Arg
*TRPV4*	(NM_021625.4)	1	c.557G>A	p.Arg186Gln
Total		51		

Legend (based on HGVS recommendations)

Two changes in one gene on different chromosomes (e.g., in recessive diseases) are shown as for example p.[Arg37Pro];[Arg37Pro]; this describes two changes in trans (derived from a gene on each chromosome (one paternal, one maternal)

*Termination codon

**Fig. 3:**
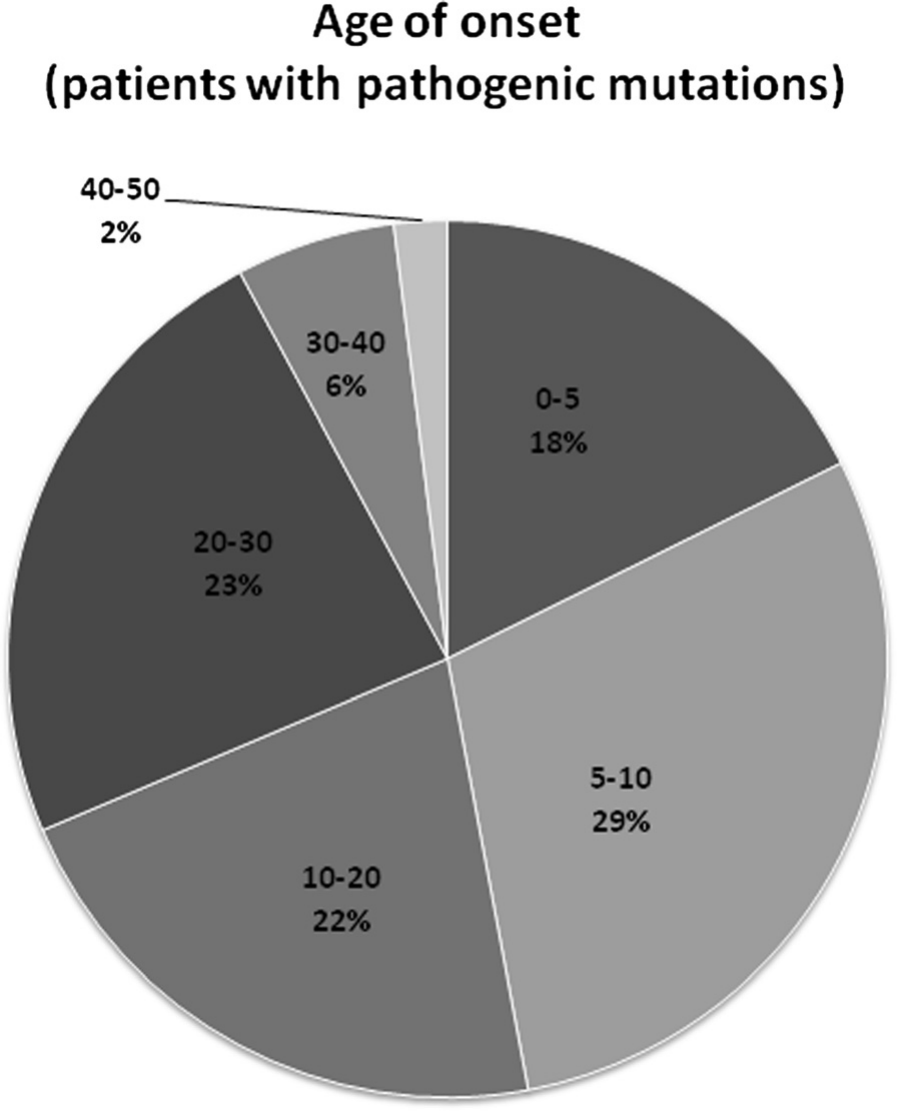
Age of onset of the disease (patients with pathogenic mutations). Legend: The graph represents the age of onset of the disease for 51 patients with causal pathogenic mutations found in the study. Each block in the graph represents a different age of onset group; age groups are described as intervals in years, and percentage of probands with onset is shown. Almost 70 % of causal mutations were found in patients with early onset (before the age of 20), only 30 % of mutations were found in patients with onset of the disease in the third life decade or later

From 51 clarified patients with causal pathogenic mutations the age of onset of the disease was in the first or second decade in 70 % of them. The age of onset after 30 years was associated with only a very low chance for clarification. This is represented in Fig. 3.

Some of the variants are described in detail below. We decided not to include detailed description for pathogenic variants which are common and clinical phenotype is presented in great detail elsewhere [11–16].

#### *AARS* (NM_001605.2): c.986G>A

Mutation p.Arg329His (c.986G>A) was found in three families with IPN. This mutation has been previously described in a large French family [11]. Segregation of the mutation has been tested. Pedigrees are shown in Additional file 1: Part 5.

Clinically, the family 826 presented with axonal neuropathy with autosomal dominant inheritance. The index patient’s (V/4) examination is presented in Additional file 1: Part 5.

The patient was re-examined recently (at the age of 41). Very slow progression of the disease was observed.

#### *DYNC1H1* (NM_001376.4): c.1792 C>T

A mutation p.Arg598Cys (c.1792 C>T) in *DYNC1H1* gene was found in two originally independent patients with early infantile onset of HMN or dSMA which later turned out to be closely related but having different surnames. The phenotype of the patient is compatible with SMALED (SMA with lower limb predominance) since the weakness on lower limbs is severe, but the upper limbs are almost unaffected and even the EMG examination was normal on upper limbs in some of the patients. The mutation was rated as causal with high probability. In the meantime, the mutation was also described in a young patient with similar phenotype [17].

#### *GJB1* deletion

A large deletion of the *GJB1* gene was found by our CNV analysis in one patient with progressive peripheral motor and sensory neuropathy. The deletion of the entire *GJB1* was later also confirmed by Multiplex Ligation-dependent Probe Amplification (MLPA, MRC-Holland, NL). The SALSA MLPA P129-B1 *GJB1* probemix was used. The patient presented with classical CMTX1 phenotype, but what was atypical for CMTX1 is the sporadic occurrence in the family due to a de-novo mutation.

#### *NDRG1* (NM_001135242.1): c.442 C>T

A homozygous mutation p.Arg148* in the *NDRG1* gene was surprisingly detected in a five years old patient with no obvious Roma origin. The mutation in heterozygous state was also detected in the father, but surprisingly not in the mother. A complete isodisomy of chromosome eight of paternal origin was confirmed with other methods. The proband is a homozygote for the mutation due to a paternal uniparental isodisomy and the diagnosis of HMSN-Lom (CMT4D) has been stated [18].

Even more surprising was that the patient is also a heterozygous carrier of the mutation p.Arg113* in the *REEP1* gene (NM_022912.2). This mutation has been already described as pathogenic for hereditary spastic paraplegia [19]. However, a segregation analysis showed that a healthy father and a healthy grandfather of the proband are also heterozygous carriers of this variant. Both of them deny having any sort of neurological problems and were also neurologically examined with normal gait, normal reflexes and no weakness. It has been therefore concluded that this variant is not causal in this particular family and may be a harmless variant.

#### *SETX* (NM_015046.5): c.[1656G>T(;)1658C>T]

A patient with progressive distal, but also proximal muscle weakness was reffered to our department when he was 11 years old. No other family members are affected. Muscle weakness, mainly on lower limbs, was noticeable from preschool age and is progressive. At examination at the age of 13 he presented with pronounced proximal and distal muscle weakness, muscle atrophies but also brisk reflexes, what is sometimes seen in HMN patients. He has problems with running and climbing. He is not able to squat. Electrophysiology revealed axonal polyneuropathy predominantly on motor nerve fibres compatible with HMN or dSMA.

The mutation c.[1656G>T(;)1658C>T] in *SETX* gene was detected. It was not detected in patient’s parents. We concluded the mutation arose de-novo and causes change NM_015046.5>c.1656_1658delCTGinsTTT on one allele of the *SETX* gene. The indel mutation causes the loss of 2 residues and the insertion of HisPhe (p.Gln552_Ser553delinsHisPhe). This is an in-frame mutation, however due to the change of two aminoacids we concluded the mutation is causal for ADSMA in this patient. De-novo origin and patient’s phenotype further support this hypothesis. No variants have been reported at this position of the *SETX* gene in various databases (EVS, dbSNPbuild137, ESP, dbSNP ShortVariants/Swiss Prot Variants).

### Possibly pathogenic variants (further studies needed)

These are novel variants in known IPN genes (Table 2). Reasons that support or oppose their pathogenic character are presented in the Additional file 1: Part 6.

**Table 2:** Novel variants in known IPN genes that are considered to be possibly pathogenic variants

Gene	Ref. sequence	Variations at DNA-level (relative to coding DNA sequence)	Variation at protein level (deduced)	Pathogenicity predictions	Locus conservation	ExAC (0.3.1) allele frequency
*AARS*	(NM_001605.2)	c.503 C>T	p.Pro168Leu	SIFT:D MT: DC	N:highly AA: highly	All: T = 0.0017 %
*BICD2*	(NM_001003800.1)	c.1540G>A	p.Gly514Ser	SIFT:D MT: DC	N:M AA:highly	All: A = 0.019 %
*DCTN1*	(NM_004082.4)	c.487G>A	p.Ala163Thr	SIFT:T MT: DC	N:W AA:Highly	All: A = 0.0029 %
*DNM2*	(NM_001005360)	c.890G>T	p.Arg297Leu	SIFT:D MT:DC	N:highly AA:highly	All: T = 0.0016 %
*DNM2*	(NM_001005360)	c.796C>T	p.Arg266Trp	SIFT:D MT:DC	N:W AA:highly	All: T = 0.0017 %
*GNB4*	(NM_021629.3)	c.125G>A	p.Arg42Gln	SIFT:D MT:DC	N:highly AA:highly	All: A = 0.0033 %
*ITPR3*	(NM_002224.3)	c.3190A>G	p.Met1064Val	SIFT:D MT:DC	N:highly AA:highly	No
*LRSAM1*	(NM_138361.5)	c.1298C>T	p.Ser433Leu	SIFT:T MT:DC	N:W AA:M	No
*PDK3*	(NM_001142386.2)	c.218A>G	p.Asn73Ser	SIFT:T MT:DC	N:W AA:M	No
*SETX*	(NM_015046.5)	c.5825T>C	p.Ile1942Thr	SIFT:D MT:DC	N:M AA:highly	All: C = 0.00084 %
*SPTLC2*	(NM_004863.3)	c.1313del	p.Cys438Leu fs*5	frameshift		No

Legend: Data were analyzed using software: Alamut Visual version 2.8 (Interactive Biosoftware, Rouen, France)[2016-07-21]

SIFT- *D* deleterious, *T* tolerated

*MT* Mutation Taster, *DC* disease causing

*PP2* PolyPhen2, *B* benign

Conservation: *N* nucleotide, *AA* amino acid; *M* moderate, *W* weakly

### Likely benign variants

That are either listed in HGMD or are otherwise interesting, but turned out to be rare benign variants are presented in Additional file 1: Part 7.

An extensive list of likely benign variants from our database is presented in Additional file 1: Part 8. These variants from our database were present in more than five samples and are not present in Human Gene Mutation Database (HGMD® Professional, BioBase International, Qiagen Company, MA, USA) and thus are considered to be rare benign variants in our population (= variants present in more than 5 alleles in 198 patients = 6/396 = population frequency 1,51 % or more).

## Discussion

Gene panel MPS enables testing of all yet known IPN genetic causes, even very rare ones, at once in parallel, with high coverage and low price per gene. Therefore gene panel testing is truly the method of choice [20, 21] for unclarified patients.

A recent study by INC – Inherited neuropathy consortium [22] has shown interesting data. In summary, we have observed a similar pattern in the distribution of genetic causes of IPN. The common CMT subtypes (*PMP22*dup/del, *GJB1, MFN2, MPZ*) account for the vast majority of clarified causes, 89.2 % of causes in INC group, 90,5 % of causes in our cohort. This result shows that testing of clinically selected patients for the relevant four most common causes is able to identify the molecular genetic cause of inherited neuropathy in approximately 90 % of patients in whom we are able to identify the cause with current knowledge and methodology. This result also shows us that the rarer types are, in reality, very or extremely rare, and in summary, that mutations in all other known IPN genes represent only approximately 10 % of yet known causes.

Our study is unique in several aspects such as the number of patients tested (one hundred and ninety-eight patients) and also in the methods used (targeted gene panel resequencing with HaloPlex custom design kits provided by Agilent was used). There are only a few similar reports published to date regarding the results from gene panel testing. Moreover, the utility of redesigning the panel regularly according to current knowledge has also been shown. Our results demonstrate that mutations in genes only recently described and newly included into the panel are responsible for more than 10 % of causes of inherited neuropathies which were aetiologically clarified in this study. Moreover, in our study we have shown, that gene panel testing is a useful tool for rare and unexpected causes of IPN, where the gene by gene approach would only hardly detect the causal mutation. Old methods (MLPA and Sanger sequencing of individual genes) were able to identify causal variants in more than 97 % of all patients (920 - before panel) and accordingly, gene panel testing enabled us to identify further 3 % of causal mutations (51- with the panel). However, this is a very important contribution of the panel. We expect that most of these 51 clarified patients would not have been clarified without the gene panel. The causes are extremely rare or unexpected due to various reasons and by single gene testing the chance for clarification would be very low. On the other hand, our results demonstrate that the use of a gene panel is clearly not rational as the first or even second test.

Through targeted gene panel sequencing many interesting findings were revealed:

- *Patient with HMSN-Lom with a homozygous mutation in the NDRG1*
  Findings such as complete isodisomy of the whole chromosome could not be expected. This case illustrates the posibillities of targeted gene panel sequencing. The proband would not have been tested for this specific Roma mutation with Sanger sequencing, because it would not have been suspected and the diagnosis could have been missed.
- *Patient with GJB1 gene deletion*
  This patient was planned for testing with Sanger sequencing of the *GJB1* gene. With routinely used PCR primers for amplification of exon 2 of the GJB1 gene a PCR product of similar length than would be expected was obtained, but it was not possible to sequence this product. Afterwards, targeted gene panel resequencing with HaloPlex was performed. From generated bam. files, almost zero coverage was observed for the *GJB1* gene region.

The deletion of the entire *GJB1* was later confirmed also by MLPA (Salsa P-0129, MRC-Holland, Amsterdam, the Netherlands). We also send the patient’s DNA sample for SNP microarray testing. The results confirmed the deletion of the Xq13.1 region including the region of the *GJB1* gene in the patient, but not in his mother. Large deletions of the region have already been described [23].

This example illustrates the ability of gene panel massively parallel sequencing to detect also larger copy number variations, especially in the hemizygous or homozygous state.

### Ragarding the mutation in *SETX* (NM_015046.5):p.[(Gln552His(;)Ser553Phe)], c.[1656G>T(;)1658C>T]

We consider this mutation to be causal in our patient. It is interesting to highlight some features of the clinical phenotype, especially brisk reflexes. Other patients described in the literature presented wih similar symptoms. Our patients is only 15 years old. At this age, brisk reflexes are described in HMN patients. Later in the course of the disease, reduced/absent reflexes are described. Brisk reflexes might be a transient phenomenon in the timeline of this disease.

There is a discussion about classification of this phenotype. It might be true that ALS4 and ADSMA are terms related to a similar phenotype, but at different times, however, some authors prefer to distinguish these two entities. We think that our patient fits best the phenotype described by Sabine Rudnik-Schoneborn as ADSMA [24].

#### Old mistakes

Targeted gene panel resequencing also revealed some old “mistakes”. A representative example is a patient with mutation p.Glu208Lys in the *GJB1* gene. This patient was previously tested for *GJB1* mutations in the year 2002, but no mutation was found at that time. Unfortunately, this was caused by a sample or PCR product exchange by a laboratory technician. Gene panel testing is thus also a useful cross-check for all previous Sanger sequencing tests.

### Mutations in common IPN genes

The high proportion of patients with mutations in common IPN genes (*PMP22* – 2 patients, *GJB1 –* 5 patients*, MFN2 –* 9 patients) detected in this study might also be surprising. These genes are widely routinely tested in our lab in relevant patients. However, these patients were not tested for mutations in these genes mainly because the clinical information we obtained was misleading or incomplete. For some of those patients the type of neuropathy was misclassified (demyelinating vs. axonal), for some patients the type of inheritace in the family (autosomal dominant vs. autosomal recessive) was misclassified. To conclude, targeted gene panel resequencing is not only able to identify mutations in rare genes, but is also a powerful tool for searching for mutations in common IPN genes. It certainly might happen that some of the patients enter genetic testing with improper or insufficient clinical data. These patients are then tested for mutations in different genes than would be appropriate. Not only are such tests useless but also the diagnostic process is hindered for a long time. This experience further supports the urgent need for as much clinical data as possible on one side and the need for effective genetic testing with careful clinical evaluation before testing on the other.

### The utility of redesigning the panel every six months

In our approach, we have developed our own custom designed gene panel. There were five respective designs, updated approximately every six months. The first gene panel design consisted of 59 genes, the second of 64, the third of 69, the fourth of 78 genes and the fifth of 93 genes.

From new genes that were just added, mutations were found in a significant proportions of them, pathogenic mutations were found in genes *ATP7A, COX6A1* and *DYNC1H1*. Possibly pathogenic variants were found in four other genes: *BICD2, GNB4, ITPR3* and *PDK3.* These results show that mutations in genes only recently described in IPN, may be responsible for more than 10 % of causes of inherited neuropathies which were etiologically clarified in this study.

It may be useful to retest unclarified patients on subsequent panels. However, during this study, this has not been done, yet. Patients in this study were tested only once with one panel design. Performing new testing for negative patients may bring out new information. Retesting by a panel is used only in selected patients and we recommend it is considered against the possibility of whole exome sequencing (WES) depending on the individual situation.

## Conclusions

In our study we have shown that targeted gene panel MPS is a powerful tool in DNA testing of IPN. In a carefully preselected cohort of patients we were able to identify the cause of IPN in 26 % of patients. A substantial part of the patients may have been detected by classical methods, single gene Sanger sequencing, if all necessary information about the patients and all previous processes have been correct.

In one-sixth of the patients (16 %) the results were inconclusive, mostly because a likely pathogenic variant in known IPN gene was found. In most of the tested genes, causal mutations were found in only a single patient or family, and only in twelve genes out of 93 were causal mutations found in two or more independent patients.

Gene panel enables testing of all yet known IPN causes in parallel with high coverage and at an affordable cost. It is therefore truly the method of choice for patients unclarified by previous testing of the most common and relevant causes of IPN.

## Additional file

Additional file 1: Part 1: Genes tested prior to this study with Sanger sequencing. Part 2: A list of genes included in consecutive designs. Part 3: Galaxy pipeline parameters and pipeline resources [25–31]. Part 4: Minimum requirements for a variant to be considered as variant of interest. Part 5: Patients with mutations in known IPN genes: Clinical data [24, 32, 33]. Part 6: Patients with novel variants in known IPN genes. Part 7: Likely Benign Variants. Part 8: Table of rare benign variants in our population = variants present in more than 5 alleles out of 306 (153 patients). (PDF 1297 kb)

## Abbreviations

CMT: Charcot-Marie-Tooth
IPN: Inherited peripheral neuropathies
WES: Whole exome sequencing
