# Supplementary material for: Improving diagnosis of inherited peripheral neuropathies through gene panel analysis
Source: Orphanet J Rare Dis. 2016 Aug 22;11(1):118. doi: 10.1186/s13023-016-0500-5 (PMC4994270; doi:10.1186/s13023-016-0500-5)
Supplement: Additional file 1: — Part 1: Genes tested prior to this study with Sanger sequencing. Part 2: A list of genes included in consecutive designs. Part 3: Galaxy pipeline parameters and pipeline resources [25–31]. Part 4: Minimum requirements for a variant to be considered as variant of interest. Part 5: Patients with mutations in known IPN genes: Clinical data [24, 32, 33]. Part 6: Patients with novel variants in known IPN genes. Part 7: Likely Benign Variants. Part 8: Table of rare benign variants in our population = variants present in more than 5 alleles out of 306 (153 patients). (PDF 1297 kb) [file 13023_2016_500_MOESM1_ESM.pdf]

SUPPLEMENTARY FILE PART 1:

In the study cohort, 47% of patients were referred as HMSN II, 30% of patients as HMSN I, 4% as having intermediate HMSN and the remaining 19% of patients was not classified.

Most patients were sporadic cases, autosomal dominant inheritance was described for 29% of patients and autosomal recessive inheritance for 7% of patients in the study cohort.

Figure A represents genes, that were tested previously Sanger sequenced in patients included in the study with no causal mutations found.

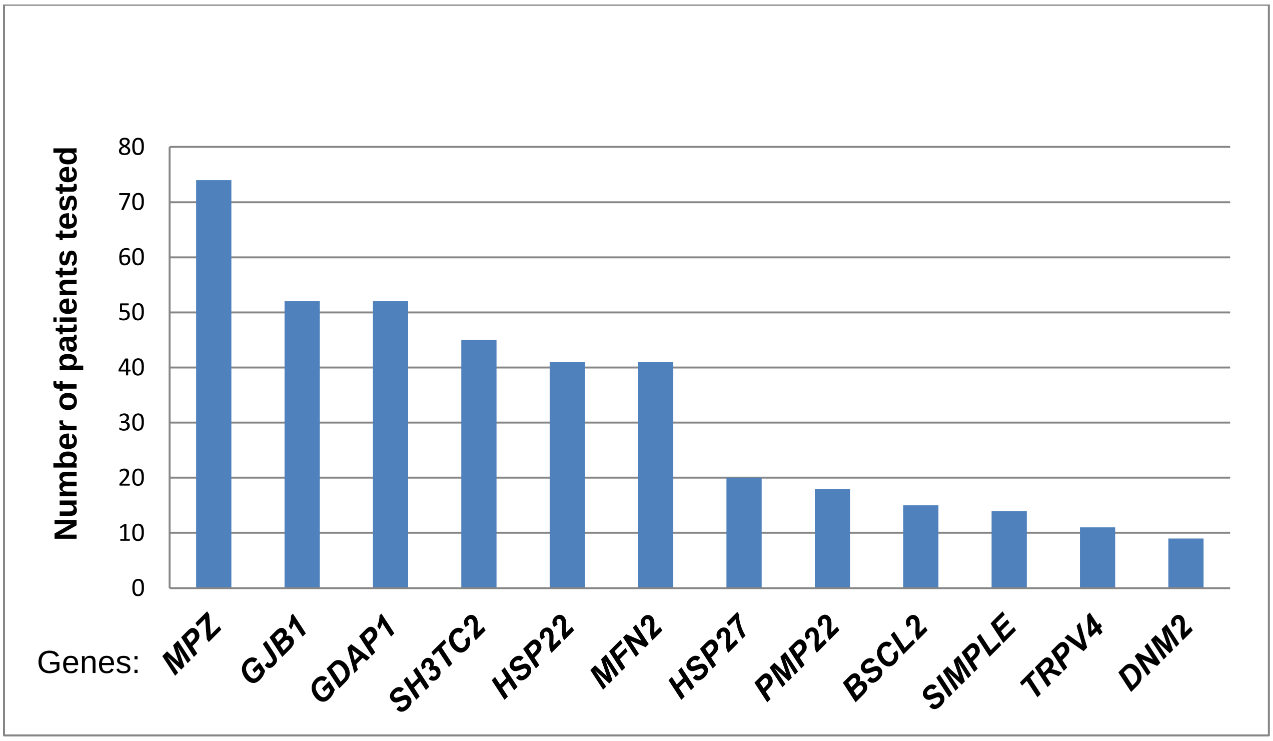

FIGURE A: GENES TESTED PRIOR TO THIS STUDY with Sanger sequencing

Legend: X – axis: genes; Y – axis: number of patients from the study cohort (total number of patients 153) tested for mutations in these genes.

SUPPLEMENTARY FILE PART 2: A list of genes included in consecutive designs

**Design 06/2013**	**Design 10/2013**	**Design 05/2014**	**Design 10/2014**	**Design 08/2015**		**Design 06/2013**	**Design 10/2013**	**Design 05/2014**	**Design 10/2014**	**Design 08/2015**
**59 genes**	**64 genes**	**72 genes**	**78 genes**	**93 genes**		**59 genes**	**64 genes**	**72 genes**	**78 genes**	**93 genes**
AARS	AARS	AARS	AARS	AARS		LITAF	LITAF	LITAF	LITAF	LITAF
	AIFM1			AIFM1		LMNA	LMNA	LMNA	LMNA	LMNA
ARHGE	ARHGE	ARHGE	ARHGE	ARHGE		LRSAM	LRSAM	LRSAM	LRSAM	LRSAM
ATL1	ATL1	ATL1	ATL1	ATL1			MARS	MARS	MARS	MARS
		ATP7A	ATP7A	ATP7A		MED25	MED25	MED25	MED25	MED25
		BICD2	BICD2	BICD2		MFN2	MFN2	MFN2	MFN2	MFN2
BSCL2	BSCL2	BSCL2	BSCL2	BSCL2		MICAL1	MICAL1	MICAL1	MICAL1	MICAL1
CCT5	CCT5	CCT5	CCT5	CCT5		MPZ	MPZ	MPZ	MPZ	MPZ
				CHCH						MT-
			COX6A	COX6A		MTMR2	MTMR2	MTMR2	MTMR2	MTMR2
CTDP1	CTDP1	CTDP1	CTDP1	CTDP1				MYH14	MYH14	MYH14
DCTN1	DCTN1	DCTN1	DCTN1	DCTN1		NDRG1	NDRG1	NDRG1	NDRG1	NDRG1
		DNAJB	DHTKD	DHTKD		NEFL	NEFL	NEFL	NEFL	NEFL
			DNAJB	DNAJB		NGF	NGF	NGF	NGF	NGF
DNM2	DNM2	DNM2	DNM2	DNM2		NTRK1	NTRK1	NTRK1	NTRK1	NTRK1
		DNMT1	DNMT1	DNMT1			PDK3	PDK3	PDK3	PDK3
				DRP2		PLEKH	PLEKH	PLEKH	PLEKH	PLEKH
				DST		PMP22	PMP22	PMP22	PMP22	PMP22
		DYNC1	DYNC1	DYNC1		PRPS1	PRPS1	PRPS1	PRPS1	PRPS1
EGR2	EGR2	EGR2	EGR2	EGR2		PRX	PRX	PRX	PRX	PRX
FAM13	FAM13	FAM13	FAM13	FAM13		RAB7A	RAB7A	RAB7A	RAB7A	RAB7A
FBLN5	FBLN5	FBLN5	FBLN5	FBLN5		REEP1	REEP1	REEP1	REEP1	REEP1
				FBXO3					SBF1	SBF1
FGD4	FGD4	FGD4	FGD4	FGD4		SBF2	SBF2	SBF2	SBF2	SBF2
FIG4	FIG4	FIG4	FIG4	FIG4						SCN11
GAN	GAN	GAN	GAN	GAN						SCN9A
GARS	GARS	GARS	GARS	GARS		SEPT09	SEPT09	SEPT09	SEPT09	SEPT9
GDAP1	GDAP1	GDAP1	GDAP1	GDAP1		SETX	SETX	SETX	SETX	SETX
GJB1	GJB1	GJB1	GJB1	GJB1					SH3BP	SH3BP
				GJB3		SH3TC	SH3TC	SH3TC	SH3TC	SH3TC
			GNB4	GNB4						SIGMA
HARS	HARS	HARS	HARS	HARS		SLC12A	SLC12A	SLC12A	SLC12A	SLC12A
HINT1	HINT1	HINT1	HINT1	HINT1		SLC18A	SLC18A	SLC18A	SLC18A	SLC18A
	HK1	HK1	HK1	HK1		SLC5A7	SLC5A7	SLC5A7	SLC5A7	SLC5A7
HSPB1	HSPB1	HSPB1	HSPB1	HSPB1						SOD1
HSPB3	HSPB3	HSPB3	HSPB3	HSPB3		SOX10	SOX10	SOX10	SOX10	SOX10
HSPB8	HSPB8	HSPB8	HSPB8	HSPB8		SPTLC	SPTLC	SPTLC	SPTLC	SPTLC
				IFRD1		SPTLC	SPTLC	SPTLC	SPTLC	SPTLC
IGHMB	IGHMB	IGHMB	IGHMB	IGHMB			SURF1			SURF1
IKBKA	IKBKA	IKBKA	IKBKA	IKBKA		TFG	TFG	TFG	TFG	TFG
INF2	INF2	INF2	INF2	INF2					TRIM1	TRIM2
			ITPR3	ITPR3		TRPV4	TRPV4	TRPV4	TRPV4	TRPV4
KARS	KARS	KARS	KARS	KARS						TUBB3
		KIF1A	KIF1A	KIF1A						VAPB
KIF1B	KIF1B	KIF1B	KIF1B	KIF1B					VCP	VCP
			KLHL1	KLHL1		WNK1	WNK1	WNK1	WNK1	WNK1
						YARS	YARS	YARS	YARS	YARS

SUPPLEMENTARY FILE PART 3: Galaxy pipeline parameters and pipeline resources

THE SCHEMATIC REPRESENTATION OF THE GALAXY PIPELINE

Legend: ROD file – regions of interest file; DOC – Depth of coverage, filter 8% - all variants that are present in population with frequency 8% and more were filtered out.

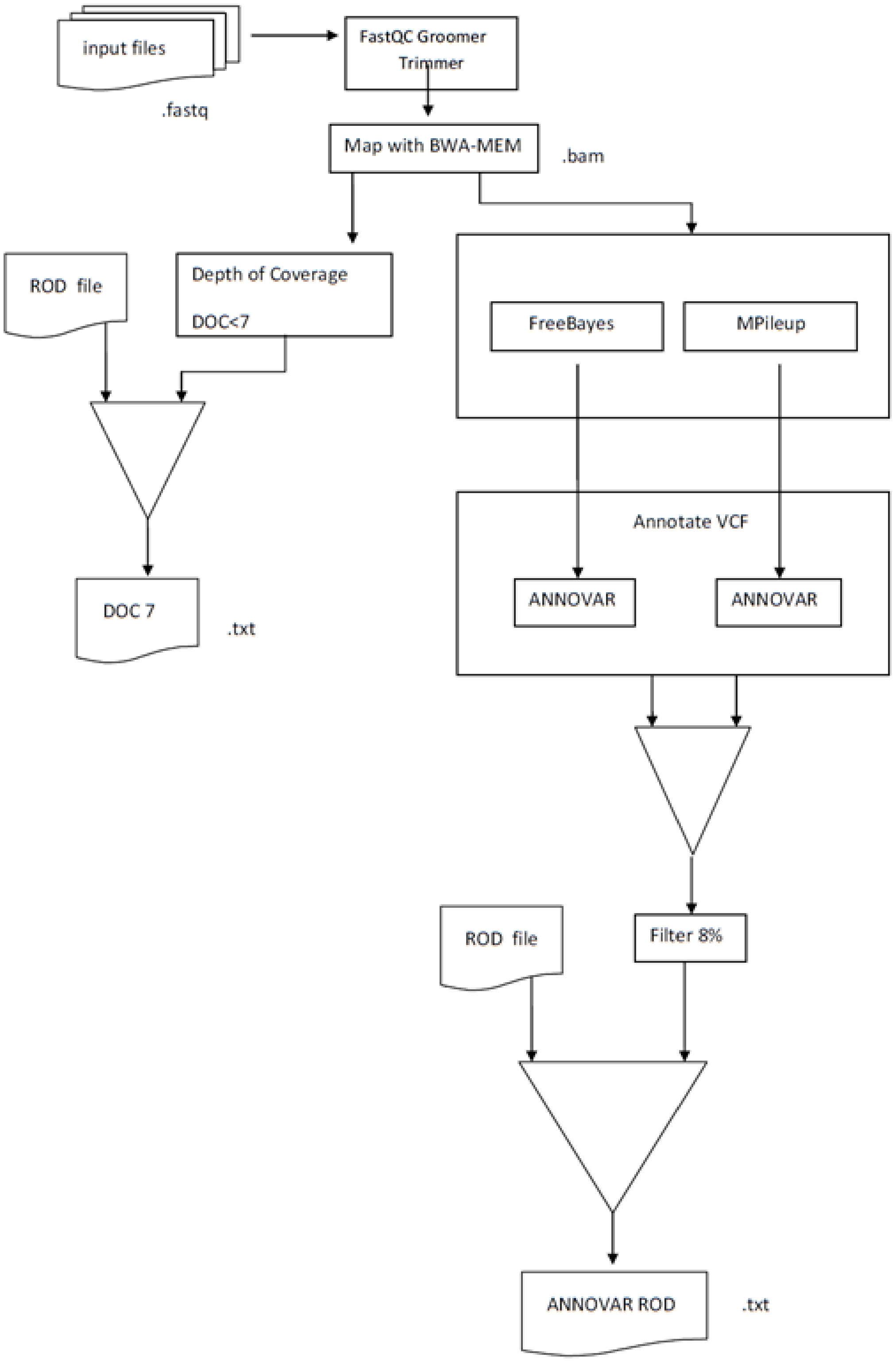

FASTQ Groomer (version 1.0.4) (Blankenberg, et al., 2010)

**Input Parameter**	**Value**
Input FASTQ quality scores type	Sanger & Illumina 1.8+
Advanced Options	advanced
Output FASTQ quality scores type	Sanger (recommended)
Force Quality Score encoding	ASCII
Summarize input data	Summarize Input

Map with BWA-MEM (version 0.8.0) (Li, 2013)

**Input Parameter**	**Value**
BWA settings to use	full
Minimum seed length (-k)	19
Band width for banded alignment (-w)	100
Off-diagonal X-dropoff (-d)	100
Look for internal seeds inside a seed longer than the minimum seed length times this value (-r)	42125
Skip seeds with more occurrences than this value (-c)	10000
Skip mate rescue (-S)	False
Skip pairing (-P)	False
Score for a sequence match (-A)	1
Penalty for a mismatch (-B)	4
Gap open penalty (-O)	6
Gap extension penalty (-E)	1
Penalty for clipping (-L)	5
Penalty for an unpaired read pair (-U)	17
Minimum score to output (-T)	30
Output all found alignments for single-end or unpaired paired-end reads (-a)	False
Mark shorter split hits as secondary (-M)	False
Specify the read group for this file? (-R)	yes
Read group identifier (ID). Each @RG line must have a unique ID. The value of ID is used in the RG tags of alignment records. Must be unique among all read groups in header section.	
Platform/technology used to produce the reads (PL)	ILLUMINA
Library name (LB)	
Sample (SM)	
Platform unit (PU)	
Sequencing center that produced the read (CN)	
Description (DS)	
Date that run was produced (DT)	
Flow order (FO). The array of nucleotide bases that correspond to the nucleotides used for each flow of each read	

The array of nucleotide bases that correspond to the key sequence of each read (KS)	
Programs used for processing the read group (PG)	
Predicted median insert size (PI)	
Suppress the header in the output SAM file	False

Depth of Coverage (version 0.0.7) (DePristo, et al., 2011)

Using reference genome	hg19
RefSeq Rod	No dataset
Partition type for depth of coverage	sample
Output format	table
Basic or Advanced GATK options	advanced
How strict should we be in validating the pedigree information	STRICT
Genomic intervals	ROD file
Interval set rule	UNION
Type of reads downsampling to employ at a given locus	NONE
Type of BAQ calculation to apply in the engine	OFF
BAQ gap open penalty (Phred Scaled)	40.0
Use the original base quality scores from the OQ tag	False
Value to be used for all base quality scores, when some are missing	0
How strict should we be with validation	STRICT
Interval merging rule	ALL
Disable experimental low-memory sharding functionality.	False
Makes the GATK behave non deterministically, that is, the random numbers generated will be different in every run	False
Fix mis-encoded base quality scores. Q0 == ASCII 33 according to the SAM specification, whereas Illumina encoding starts at Q64. The idea here is simple: we just iterate over all reads and subtract 31 from every quality score.	False
Basic or Advanced Analysis options	basic

FreeBayes (0.9.14) (Garrison and Marth, 2012)

Input Parameter	Value
Load reference genome from	history
Use the folloing dataset as the reference sequence	hg19.fasta
Limit variant calling to a set of regions?	do_not_limit
Choose parameter selection level	simple

MPileup (version 0.0.2) (Li, 2011a; Li, 2011b; Li, et al., 2009);

Input Parameter	Value
Choose the source for the reference list	history
Using reference file	hg19.fasta
Genotype Likelihood Computation	perform_genotype_likelihood_computation
Phred-scaled gap extension sequencing error probability	20
Coefficient for modeling homopolymer errors.	100
Perform INDEL calling	perform_indel_calling
Skip INDEL calling if the average per-sample depth is above	250
Phred-scaled gap open sequencing error probability	40
Set advanced options	advanced
Do not skip anomalous read pairs in variant calling	False
Disable probabilistic realignment for the computation of base alignment quality (BAQ)	True
Coefficient for downgrading mapping quality for reads containing excessive mismatches	0
Max reads per BAM	250
Extended BAQ computation	True
List of regions or sites on which to operate	No dataset
Minimum mapping quality for an alignment to be used	0
Minimum base quality for a base to be considered	13
Only generate pileup in region	
Output per-sample read depth	False
Output per-sample Phred-scaled strand bias P-value	False

ANNOVAR Annotate VCF (version 0.1)

Input Parameter	Value
Gene Annotations	
Annotation Regions	refGene ensGene knownGene dgvMerged

Annotation Databases	avsift ljb_sift ljb2_sift ljb26_sift ljb_pp2 ljb2_pp2hdiv ljb2_pp2hvar ljb26_pp2hvar ljb2_phylop ljb23_phylop ljb26_phylop100way_vertebrate ljb23_lrt ljb26_lrt ljb26_mt ljb26_ma ljb26_fathmm ljb26_siphy ljb26_gerp++ ljb26_metasvm ljb26_metalr ljb26_vest ljb26_cadd ljb26_all cg46 cg69 cosmic70 esp6500si_all exac01 AFR.sites.2014_10 ALL.sites.2014_10 AMR.sites.2014_10 EAS.sites.2014_10 EUR.sites.2014_10 SAS.sites.2014_10 snp129 snp138 snp130NonFlagged snp138NonFlagged clinvar_20131105 clinvar_20140929 popfreq_max popfreq_all gerp++elem gerp++gt2 caddgt20 caddgt10

Filter (version 1.1.0)

esp5400_ea, esp5400_all, esp6500si_all < 8%

SUPPLEMENTARY FILE PART 4: Minimum requirements for a variant to be considered as variant of interest.

**Tool**	**Specifications**	**Minimum requirements to be graded as variant of interest**
**Effect of the mutation**	Nonsense mutations,<br>Missense mutations and<br>Synonymous variants<br>Splice variants	Nonsense variants – most interesting<br>Missense variants - carefully evaluated with all other tools,<br>Synonymous variants – only if previously reported as pathogenic OR if algorithms predict alternative splicing, otherwise not evaluated<br>Splice variants – variants ±1-2bp most interesting, variants as much as ±10bp are also evaluated
**Conservation score**	PhyloP, GERP	At least some evidence of conservation – no negative values - Phylo P> 0 a GERP > 0
**Prediction tools**	Mutation Taster, PolyPhen2, SIFT	At least one deleterious prediction
**Population frequency**	EVS, ExAC and 1000 genomes	less than 0.005% for AD,<br>less than 2% for AR,
**Population frequency in our population**	In-house database of variants	present in less than 3 alleles in one sequencing run (48 samples) AND present in 5 alleles or less than 5 alleles from 198 patients

SUPPLEMENTARY FILE 5:

PATIENTS WITH MUTATIONS IN KNOWN IPN GENES: CLINICAL DATA

FIGURE 1 : PEDIGREES

Panel A - Pedigrees of IPN families with *AARS* (NM_001605.2):p.Arg329His (c.986 G>A) mutation

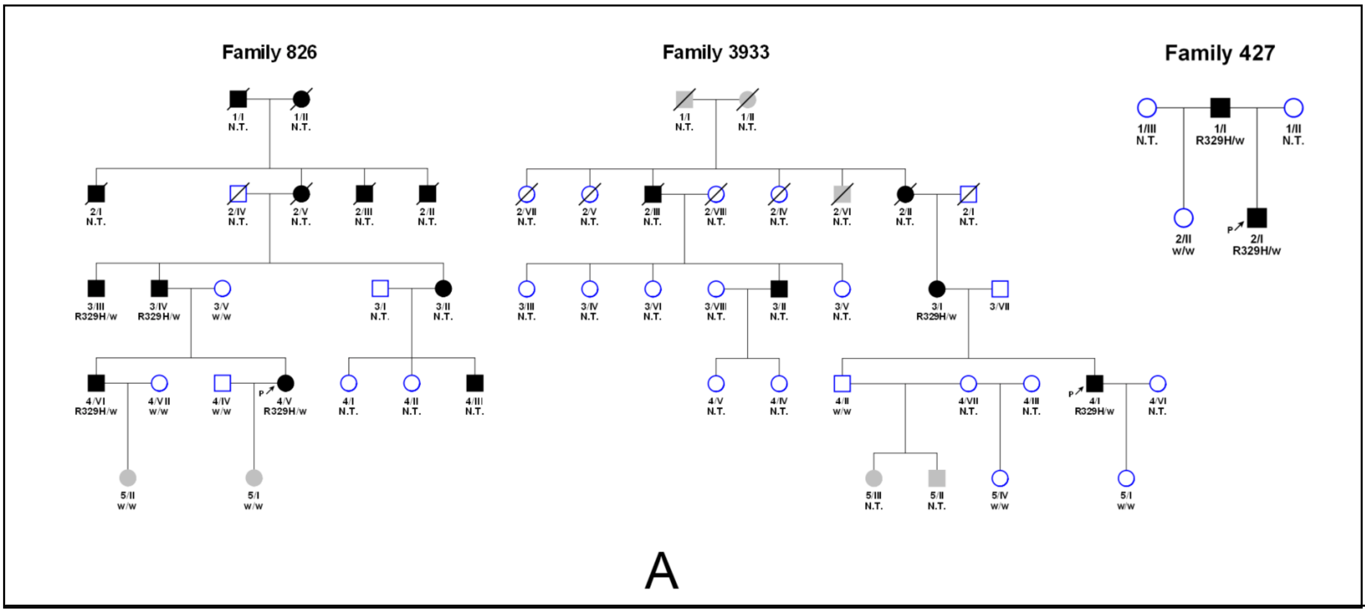

Legend: **square**: male; **circle**: female; **blacked filled symbol**: affected; **empty symbol**: unaffected; **slashed symbol**: desceased; **w**. – wild type; **N.T**. – not tested.; **R329H** - *AARS* (NM_001605.2):p.Arg329His (c.986 G>A) mutation;

FIGURE 2: PATIENT 4/V FROM FAMILY 826 AT THE AGE OF 41 YEARS

Patient 4/V from family 826 with *AARS* mutation p.Arg329His at the age of 41. The most prominent feature is plegia of toes. Genua recurvata and Achill. tendon shortenings (to 90°) and absent tendon reflexes in all limbs were also observed.

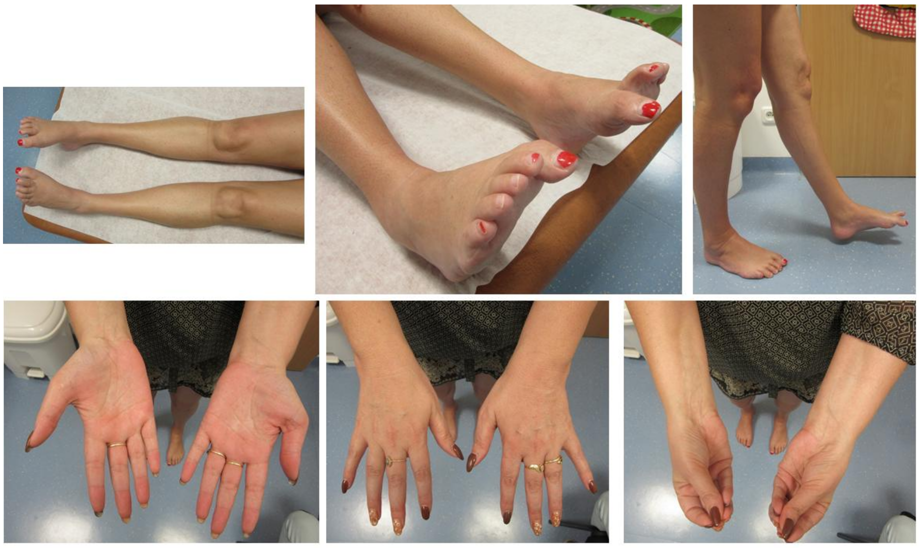

TABLE 1: PATIENT 4/V FROM FAMILY 826 AT THE AGE OF 31 YEARS

**826 - AARS - age at examiantion 31**	**Test**		**Results**
**Clinical course**			First symptoms at the age of ten, difficulties running, jumping…
**Upper limbs**	Muscle atrophies		No
	Contractures/ deformities		No
	Reflexes		Reflexes C5/6 are absent. Reflex C7 sin +
	Sensory testing		
		Pin prick test	N.T.
		Vibration test	Vibration test at fingers was 7/8
**Lower limbs**	Muscle atrophies		Slight atrophies distally are present
	Contractures/ deformities		Achill. tendons shortening (90°)
	Reflexes		Reflexes L2/S2 absent
	Sensory testing		Sensory loss in stockings distribution
		Pin prick test	plantum 2/10
		Vibration test	At toe tips 5/8
**Gait**			Able to walk without support, not able to stand on heels or toes
**Other**			No
**EMG test - summary**	Sensory nerve conduction studies		Sural nerve sensory response not evoked
	Motor nerve condustion studies		Normal nerve conduction velocities, but reduction in CMAP (3.5mV median nerve Left)

Supplementary file 6:

Patients with novel variants in known IPN genes (Table 2):

Clinical data

BICD2 (NM__001003800.1):p.Gly514Ser (c.1540G>A)

The patient is a sporadic case in the family, segregation analysis has not been possible, but the mutation might be causal based on information that the mutation is located in Bicaudal-D protein domain, both SIFT and Mutation Taster predictions are serious, and the variant has no population frequency score.

DCTN1 (NM_004082.4):p.Ala163Thr (c.487G>A)

This variant was detected in a male patient with axonal neuropathy. From other family members only the daugther as the only offspring of the patient was available for DNA and neurological examination. The daugther is healthy and is not a carrier of the mutation in the *DCTN1* gene. The mother of the patient is deceased, the father is unknown. A different mutation in *DCTN1* gene has been described in a paper by Puls, et al. [1]. They describe a family with autosomal dominant form of slowly progressive lower motor neuron disease without sensory symptoms. Normal sensory NCV was observed also for our patient. However, authors Puls and colleques also describe additional features, vocal cord paralysis and small hand muscle atrophy, which were not observed in our patient.

[1] Puls I, Jonnakuty C, LaMonte BH, et al. Mutant dynactin in motor neuron disease. *Nat Genet.* Apr 2003;33(4):455-456.

Table 2: A patient with *DCTN1* (NM_004082.4):p.Ala163Thr (c.487G>A) mutation at the age of 49.

4411 - DCTN1 - age 49	Test		Results
**Clinical course**			First symptoms at the age of ten,
**Upper limbs**	Muscle atrophies		No
	Contractures/ deformities		No
	Reflexes		Reflexes C5-C8 are absent.
	Muscle strenght (MRC scale - Medical Research Council scale): grades 0-5		Muscle strength is preserved.
			Shoulder abduction, elbow flexion/extension 5
			N.A
			N.A.
	Sensory testing		
		Pin prick test	
		Vibration test	Vibration test at fingers was 7/8
**Lower limbs**	Muscle atrophies		At lower limbs profound atrophies distally are present, plus hypotrophies up to midthigh - symetrically
	Contractures/ deformities		Foot deformity - pes cavus, Achill. tendons shortening (90°)
	Reflexes		Knee reflex was present, but depressed (1+), ankle reflex absent (0)
	Muscle strenght (MRC scale - Medical Research Council scale): grades 0-5		Muscle strength proximally was preserved, but not distally
			Hip flexion/extension 5
			Knee flexion/extension 5
			Foot dorsiflexion/plantar flexion 4
			Big toe extension/ toes extension 4
	Sensory testing		
		Filamentum test	plantum 2/10
		Vibration test	At toe tips 0/8, metatarsal phalangeal joint 5/8 and tibial tuberosity was 5/8.
**Gait**			Able to walk without support, not able to stand on heels or toes
**Other**			Romberg +
**EMG test - summary**	Sensory nerve conduction studies		Normal
	Motor nerve conduction studies		N.A.

GNB4 (NM_021629.3):p.Arg42Gln (c.125G>A)

The index patient from this family presented with intermediate type of CMT. The same was described in a primary report by Soong, et al.[2]. Similarly, an affected brother of the patient has the same mutation. The variant is in a highly conserved region. The SIFT and Mutation taster predictions are deleterious/disease causing. The mutation has no frequency in EVS, however, in ExAC, the mutation hes been reported in 4 alleles (out of 121000), but these were from different populations [Latino (3 alleles) and South Asian (1 allele) populations].

SETX (NM_015046.5):p.Ile1942Thr (c.5825T>C)

A variant p.Ile1942Thr was detected on both alleles of the *SETX* gene (in homozygous state). Only heterozygous mutations in the *SETX* gene have been described as a cause of HMN so far. This variant is interesting as it is highly conserved, predictions are deleterious and there is no population frequency in EVS and only 0.001% in ExAC. A brother of the patient is similarly affected. Therefore, testing him for the presence of the mutation would help to elucidate the character of the variant. However, he was not available for DNA testing and neurological reexamination. We speculate these siblings might be affected by an autosomal recessive ataxia with oculomotor apraxia caused by biallelic loss of function mutations in the *SETX* gene, but we are not able to prove this at this time.

---

[2] Soong BW, Huang YH, Tsai PC, et al. Exome sequencing identifies GNB4 mutations as a cause of dominant intermediate Charcot-Marie-Tooth disease. *Am J Hum Genet.* Mar 7 2013;92(3):422-430.

Supplementary file 7:

Likely Benign Variants

That are either listed in HGMD or are otherwise interesting, but turned out to be rare benign variants are presented in supplementary file table 3.

Gene(Ref. sequence)	Variations at DNA-level (relative to coding DNA sequence) Variation at protein level (deduced)	No. of families	Reasons why likely benign	Primary reason why interesting	Pathogenicity predictions	Locus conservation	ExAC (0.3.1) allele frequency
BICD2 (NM_001003800.1)	c.269 G>A (p. Lys90Arg)	2	Population frequency (EVS 0,58%)	Listed in HGMD as DM?;	SIFT:T<br>MT:DC	N:W<br>AA:M	All<br>G=0,47%
DNM2 (NM_001005361.2)	c.2156G>A (p.Arg719Gln)	1	rare benign polymorphism, healthy mother and a healthy brother are also carriers	Highly conserved, deleterious predictions,	SIFT:D<br>MT:DC	N:highly<br>AA:highly	No
DNM2 (NM_001005360)	c.2201A>G (p.Asn734Ser)	1	Not segregating, heatlhy mother of the patient is a carrier		SIFT:T<br>MT:DC	N:N.C.<br>AA:M	All<br>G=0,029%
DYNC1H1 (NM_001376.4)	c.239A>T (p.Glu80Val)	1	Similarly affected sister is not a carrier of the variant, the phenotype of the patient is different from what has been published for patients with DYNC1H1 mutations	Mutation Taster prediction: disease causing; no frequency data in EVS;	SIFT:T<br>MT:DC	N:M<br>AA:W	No
HARS (NM_002109.5)	c.1402 G>A (p.Glu468Lys)	1	Not segregating, the mother of the patient is a carrier of the variant, the mother is heatlhy	Novel variant in new CMT gene, without population frequency;	SIFT:T<br>MT:DC	N:M<br>AA:M	All<br>A=0,014%
HARS (NM_002109.5)	c.614G>A (p.Gly205Asp)	1	Not segregating, the healthy father of the patient is also a carrier of the mutation,		SIFT:T<br>MT:DC	N:highly<br>AA:highly	All<br>A=0,26%
HSPB1 (NM_001540.3)	c.390G>C (p.Glu130Asp)	1	Not segregating (large pedigree)		SIFT:T<br>MT:DC	N:highly<br>AA:highly	No

GARS (NM_002047.2)	c.2074 A>G (p.Met692Val)	1	Not segregating (large pedigree)		SIFT:T<br>MT:DC	N:highly<br>AA:highly	No
NEFL (NM_006158.4)	c.1585A>G (p.Lys529Glu)	1	Not segregating (large pedigree)		SIFT:T<br>PP2:B	N:W<br>AA:highly	All<br>G=0,0033%

Table 3: Likely benign variants: Novel variants in known IPN genes that turned out to be rare benign variants.

Legend: Data were analyzed using software: **Alamut Visual version 2.8 (Interactive Biosoftware, Rouen, France)[2016-07-21]**

SIFT- D:deleterious; T:tolerated

MT- Mutation Taster- DC: disease causing

PP2- PolyPhen2; B-benign

Conservation: N- nucleotide; AA-amino acid; Highly; M-moderate;W:weakly;N.C.:not conserved

SUPPLEMENTARY FILE 8: Table of rare benign variants in our population = variants present in more than 5 alleles out of 306 (153 patients)

CHR	POS	REF=> ALT	Number of families with variant	RefGene_function	RefGene_gene
1	6528468	C=>T	25	exonic	PLEKHG5
1	6528589	C=>T	13	exonic	PLEKHG5
1	6529182	TTCC=>T	18		PLEKHG5
1	6529188	C=>T	26	exonic	PLEKHG5
1	6529443	A=>G	30	exonic	PLEKHG5
1	6531124	T=>C	24	exonic	PLEKHG5
1	6531589	C=>T	14	exonic	PLEKHG5
1	6531650	C=>T	22	exonic	PLEKHG5
1	6533393	G=>C	39	exonic	PLEKHG5
1	6545390	A=>T	40	exonic	PLEKHG5
1	6550505	C=>T	11	UTR5	PLEKHG5
1	6557009	A=>C	41	intronic	PLEKHG5
1	6579521	G=>C	10	exonic	PLEKHG5
1	10271688	C=>G	12	intronic	KIF1B
1	10318652	C=>G	34	exonic	KIF1B
1	10355834	C=>T	21	intronic	KIF1B
1	10364057	A=>G	5	exonic	KIF1B

1	10364260	A=>G	6	exonic	KIF1B
1	10421878	A=>G	20	exonic	KIF1B
1	10435324	C=>A	70	exonic	KIF1B
1	12040324	A=>G	41	UTR5	MFN2
1	12040479	T=>C	50	UTR5	MFN2
1	12065841	C=>T	7	exonic	MFN2
1	33245802	C=>G	35	exonic	YARS
1	115829313	G=>A	112	exonic	NGF
1	156105028	T=>C	13	exonic	LMNA
1	156106185	T=>C	33	exonic	LMNA
1	156107534	C=>T	66	exonic	LMNA
1	156785617	G=>A	125	UTR5	NTRK1
1	156846233	G=>A	55	exonic	NTRK1
1	156848909	CA=>C	8	exonic	NTRK1
1	156848918	C=>T	18	exonic	NTRK1
1	156848946	G=>T	17	exonic	NTRK1
1	156848995	C=>T	93	exonic	NTRK1
1	161275943	C=>T	11	exonic	MPZ
2	74596527	C=>T	8	exonic	DCTN1
2	74598723	T=>C	7	exonic	DCTN1
2	86481835	C=>T	57	exonic	REEP1
2	86507135	A=>G	14	intronic	REEP1
2	108608648	A=>G	16	exonic	SLC5A7
2	198362018	T=>C	16	exonic	HSPD1
2	198363504	A=>G	21	exonic	HSPD1
2	198364518	G=>A	23	UTR5	HSPD1
2	220146651	G=>A	25	intronic	DNAJB2
2	220147847	G=>A	6	intronic	DNAJB2
2	235949877	T=>C	11	exonic	SH3BP4
2	235949920	A=>G	10	exonic	SH3BP4
2	235950002	G=>A	12	exonic	SH3BP4
2	235950187	T=>C	34	exonic	SH3BP4
2	235950284	A=>C	9	exonic	SH3BP4
2	235950391	C=>T	5	exonic	SH3BP4
2	235951819	A=>G	50	exonic	SH3BP4
2	241680802	G=>A	64	intronic	KIF1A
2	241685586	G=>A	19	exonic	KIF1A
2	241696837	CTCATCCTCC=>C	30	exonic	KIF1A
2	241696840	ATCC=>A	63	exonic	KIF1A
2	241700676	G=>A	9	exonic	KIF1A
2	241706757	T=>C	12	exonic	KIF1A
2	241713646	A=>G	70	exonic	KIF1A
2	241722445	G=>A	38	intronic	KIF1A
2	241727459	G=>C	60	intronic	KIF1A
2	241727461	T=>C	39	intronic	KIF1A

3	100467018	T=>C	100	exonic	TFG
3	128525253	C=>T	16	exonic	RAB7A
3	179137273	A=>G	38	exonic	GNB4
3	179169230	T=>G	24	UTR5	GNB4
4	154074370	CCTT=>C	19		
4	154197234	T=>G	25	exonic	TRIM2
4	154216710	G=>A	50	exonic	TRIM2
5	10250430	G=>A	140	UTR5	CCT5
5	10250443	T=>C	140	UTR5	CCT5
5	10250728	C=>G	60	intronic	CCT5
5	10254817	A=>G	129	exonic	CCT5
5	10256161	T=>C	101	exonic	CCT5
5	10258512	G=>A	13	exonic	CCT5
5	10262740	C=>A	101	intronic	CCT5
5	16478200	G=>A	76	exonic	FAM134B
5	32379210	G=>A	16	intronic	ZFR
5	32400266	A=>G	23	exonic	ZFR
5	32403346	C=>T	32	exonic	ZFR
5	53751901	G=>A	115	exonic	HSPB3
5	53751988	T=>C	121	exonic	HSPB3
5	130500751	A=>C	65	intronic	HINT1
5	130500842	A=>G	9	exonic	HINT1
5	140054257	C=>T	57	intronic	HARS
5	140057535	G=>A	30	exonic	HARS
5	148383345	C=>G	37	UTR3	SH3TC2
5	148383411	TCAA=>T	17	UTR3	SH3TC2
5	148383412	CAA=>C	40	UTR3	SH3TC2
5	148386525	T=>C	5	exonic	SH3TC2
5	148386525	T=>G	98	exonic	SH3TC2
5	148388420	C=>T	12	exonic	SH3TC2
5	148406386	T=>C	36	intronic	SH3TC2
5	148407708	A=>C	142	exonic	SH3TC2
5	148407893	C=>A	67	exonic	SH3TC2
5	148408101	A=>G	110	exonic	SH3TC2
5	148425518	A=>T	32	intronic	SH3TC2
6	33626905	G=>T	13	intronic	ITPR3
6	33626906	T=>G	6	intronic	ITPR3
6	33636907	C=>T	58	exonic	ITPR3
6	33638180	C=>T	30	exonic	ITPR3
6	33641379	T=>C	54	exonic	ITPR3
6	33643558	G=>A	37	exonic	ITPR3
6	33646328	T=>C	59	intronic	ITPR3
6	33647651	A=>G	63	intronic	ITPR3
6	33648097	A=>G	13	intronic	ITPR3
6	33648228	C=>T	69	splicing	ITPR3

6	33650430	G=>C	36	intronic	ITPR3
6	33651129	C=>T	24	exonic	ITPR3
6	33653448	C=>T	54	exonic	ITPR3
6	33653486	G=>A	13	exonic	ITPR3
6	33658780	C=>T	5	exonic	ITPR3
6	33659472	C=>G	57	exonic	ITPR3
6	109767632	T=>TGTGGTCTGGTCAGTGACCTGCCCAGG	5	intronic	MICAL1
6	109767644	C=>T	12	intronic	MICAL1
6	109767930	G=>T	67	exonic	MICAL1
6	109767931	C=>T	52	exonic	MICAL1
6	109768295	G=>A	83	exonic	MICAL1
6	109770869	G=>A	17	exonic	MICAL1
6	109771691	G=>A	5	exonic	MICAL1
6	109775366	C=>T	17	exonic	MICAL1
6	109775436	T=>C	85	exonic	MICAL1
6	110036274	T=>C	7	intronic	FIG4
6	110064928	A=>T	10	exonic	FIG4
6	110106234	A=>G	26	intronic	FIG4
6	110107517	T=>C	32	exonic	FIG4
6	110146303	G=>A	96	exonic	FIG4
7	30634630	G=>C	17	exonic	GARS
7	30634661	C=>G	132	exonic	GARS
7	30634764	C=>T	17	intronic	GARS
7	30673345	C=>T	106	intronic	GARS
7	75932038	G=>A	5	exonic	HSPB1
8	1806229	A=>C	66	exonic	ARHGEF10
8	1806288	T=>C	8	intronic	ARHGEF10
8	1806289	T=>C	8	intronic	ARHGEF10
8	1808256	A=>G	13	exonic	ARHGEF10
8	1817367	G=>A	60	exonic	ARHGEF10
8	1830794	G=>A	28	intronic	ARHGEF10
8	1833801	G=>C	13	exonic	ARHGEF10
8	1841758	T=>C	9	exonic	ARHGEF10
8	1846688	C=>T	14	exonic	ARHGEF10
8	1857548	T=>C	40	exonic	ARHGEF10
8	1857591	G=>A	9	exonic	ARHGEF10
8	1873540	C=>T	24	exonic	ARHGEF10
8	1876631	C=>T	37	exonic	ARHGEF10
8	1877480	T=>G	30	exonic	ARHGEF10
8	1900911	C=>T	28	exonic	ARHGEF10
8	1905132	G=>A	87	exonic	ARHGEF10
8	24811063	GAG=>G	6		
8	24811064	AG=>A	138	"exonic;splicing"	"NEFL;NEFL"
8	75262798	G=>C	106	exonic	GDAP1
8	75274141	T=>G	79	exonic	GDAP1

8	134260174	G=>A	49	intronic	NDRG1
8	134260948	A=>G	10	intronic	NDRG1
8	134292516	A=>G	102	intronic	NDRG1
9	35060302	T=>C	27	intronic	VCP
9	35061694	G=>GACAGTACACAA	22	intronic	VCP
9	35062972	C=>T	58	intronic	VCP
9	35071773	G=>A	6	intronic	VCP
9	35071774	C=>A	6	intronic	VCP
9	94812355	T=>C	5	intronic	SPTLC1
9	111641825	G=>A	8	exonic	IKBKAP
9	111651620	A=>T	54	exonic	IKBKAP
9	111653574	C=>G	83	exonic	IKBKAP
9	111656228	T=>A	6	exonic	IKBKAP
9	111659439	T=>C	12	exonic	IKBKAP
9	111659483	T=>G	41	exonic	IKBKAP
9	111660851	C=>T	6	exonic	IKBKAP
9	111663754	G=>A	21	exonic	IKBKAP
9	111663793	C=>T	22	exonic	IKBKAP
9	111668652	C=>T	9	exonic	IKBKAP
9	111678508	C=>T	8	exonic	IKBKAP
9	111679872	G=>A	12	exonic	IKBKAP
9	111679940	T=>C	11	exonic	IKBKAP
9	111688828	C=>T	21	exonic	IKBKAP
9	111696389	G=>C	30	UTR5	IKBKAP
9	130214414	C=>T	5	intronic	LRSAM1
9	130219669	C=>T	115	exonic	LRSAM1
9	130242109	C=>T	109	intronic	LRSAM1
9	130242166	A=>G	132	exonic	LRSAM1
9	130258319	A=>C	8	exonic	LRSAM1
9	130259618	A=>C	132	intronic	LRSAM1
9	135139826	T=>C	7	exonic	SETX
9	135139901	T=>C	76	exonic	SETX
9	135152439	A=>G	16	intronic	SETX
9	135158690	C=>T	5	exonic	SETX
9	135172412	A=>G	31	exonic	SETX
9	135173685	T=>C	41	exonic	SETX
9	135202829	T=>C	82	exonic	SETX
9	135203231	C=>T	107	exonic	SETX
9	135203409	A=>C	96	exonic	SETX
9	135203530	A=>C	6	exonic	SETX
9	135203838	G=>A	22	exonic	SETX
9	135204010	T=>C	9	exonic	SETX
9	135205006	G=>C	11	exonic	SETX
9	135206460	A=>G	77	exonic	SETX
10	50820345	C=>A	134	exonic	SLC18A3

10	50820370	C=>T	9	exonic	SLC18A3
10	64573771	C=>T	143	exonic	EGR2
10	71038431	C=>T	7	intronic	HK1
10	71060610	A=>G	102	exonic	HK1
10	71060621	C=>T	14	intronic	HK1
10	71103597	C=>G	70	exonic	HK1
10	71142420	G=>A	124	exonic	HK1
10	99498234	G=>A	46	UTR5	ZFYVE27
10	99504595	G=>A	6	exonic	ZFYVE27
10	99504630	G=>T	65	exonic	ZFYVE27
11	9812236	G=>A	76	ncRNA_intronic	SBF2-AS1
11	9853777	G=>C	15	exonic	SBF2
11	9861208	G=>C	8	exonic	SBF2
11	9879838	C=>T	25	exonic	SBF2
11	10019879	G=>A	11	exonic	SBF2
11	62458275	T=>C	50	exonic	BSCL2
11	62469929	C=>A	38	ncRNA_intronic	HNRNPUL2-BSCL2
11	62473752	C=>T	5	ncRNA_intronic	HNRNPUL2-BSCL2
11	68671419	C=>T	53	UTR5	IGHMBP2
11	68671477	T=>C	142	exonic	IGHMBP2
11	68673630	C=>T	5	exonic	IGHMBP2
11	68678962	T=>C	141	exonic	IGHMBP2
11	68682402	A=>G	53	exonic	IGHMBP2
11	68701948	C=>T	52	exonic	IGHMBP2
11	68703959	A=>G	55	exonic	IGHMBP2
11	68704028	C=>T	45	exonic	IGHMBP2
11	68704264	C=>T	83	exonic	IGHMBP2
11	68705674	C=>A	51	exonic	IGHMBP2
11	95580926	G=>A	8	exonic	MTMR2
11	95657111	T=>G	77	exonic	MTMR2
12	862989	T=>C	144	exonic	WNK1
12	863152	G=>A	22	exonic	WNK1
12	939302	A=>G	90	exonic	WNK1
12	968400	C=>A	17	intronic	WNK1
12	968489	T=>C	16	exonic	WNK1
12	970174	T=>A	15	intronic	WNK1
12	971291	C=>T	15	exonic	WNK1
12	974404	T=>C	114	exonic	WNK1
12	987482	G=>A	78	exonic	WNK1
12	987534	C=>CTT	36	intronic	WNK1
12	988894	G=>A	30	exonic	WNK1
12	990912	A=>C	125	exonic	WNK1
12	992229	C=>T	19	intronic	WNK1

12	993930	C=>T	90	exonic	WNK1
12	994014	C=>T	42	exonic	WNK1
12	994487	G=>C	144	exonic	WNK1
12	998365	G=>T	60	exonic	WNK1
12	1017197	C=>T	143	exonic	WNK1
12	32655085	C=>T	10	UTR5	FGD4
12	32687352	A=>G	5	intronic	FGD4
12	32735236	C=>T	86	exonic	FGD4
12	32755259	G=>A	21	intronic	FGD4
12	32764184	G=>A	42	exonic	FGD4
12	32777362	G=>A	37	exonic	FGD4
12	32778581	T=>C	24	intronic	FGD4
12	32791796	G=>A	20	intronic	FGD4
12	110226379	G=>A	35	exonic	TRPV4
12	110238481	G=>A	10	exonic	TRPV4
12	110238487	A=>G	18	exonic	TRPV4
12	110240838	T=>G	107	exonic	TRPV4
12	110252547	G=>A	5	exonic	TRPV4
12	110252569	C=>A	6	exonic	TRPV4
14	51054598	A=>G	27	exonic	ATL1
14	51057727	G=>A	77	exonic	ATL1
14	51062357	G=>A	30	intronic	ATL1
14	77978621	A=>C	14	UTR3	SPTLC2
14	78028803	A=>G	120	exonic	SPTLC2
14	92339908	C=>T	19	intronic	FBLN5
14	92340722	G=>A	26	intronic	FBLN5
14	92340810	G=>A	7	intronic	FBLN5
14	92340901	A=>G	18	intronic	FBLN5
14	92340992	G=>A	20	intronic	FBLN5
14	92343894	G=>A	11	exonic	FBLN5
14	92347680	A=>G	136	exonic	FBLN5
14	102446161	G=>A	7	exonic	DYNC1H1
14	102463407	A=>G	23	exonic	DYNC1H1
14	102482399	C=>T	20	exonic	DYNC1H1
14	102493761	A=>G	24	exonic	DYNC1H1
14	102504838	C=>T	9	exonic	DYNC1H1
14	102508056	C=>A	14	exonic	DYNC1H1
14	102514227	T=>C	35	exonic	DYNC1H1
14	102515015	G=>A	44	intronic	DYNC1H1
14	105167744	G=>A	6	exonic	INF2
14	105167807	C=>T	76	exonic	INF2
14	105173862	ACCCCACCCCCAC=>A	28	exonic	INF2
14	105174110	A=>C	59	exonic	INF2
14	105177351	CA=>C	110		INF2
14	105179194	T=>C	136	exonic	INF2

14	105180565	T=>C	115	exonic	INF2
14	105180652	C=>T	5	exonic	INF2
14	105180706	A=>C	85	exonic	INF2
14	105180706	A=>G	54	exonic	INF2
14	105180785	C=>T	22	exonic	INF2
15	34528948	G=>A	78	exonic	SLC12A6
15	34542872	C=>G	10	exonic	SLC12A6
15	34544468	C=>T	32	exonic	SLC12A6
15	34546704	G=>A	6	exonic	SLC12A6
15	34551082	G=>A	65	exonic	SLC12A6
16	11647492	T=>C	60	exonic	LITAF
16	11647532	C=>T	7	exonic	LITAF
16	11680148	T=>C	15	UTR5	LITAF
16	70287177	A=>G	140	exonic	AARS
16	70303580	G=>A	107	exonic	AARS
16	75674252	TAAAA=>T	104		
16	75675609	T=>C	22	exonic	KARS
16	81348764	C=>T	7	exonic	GAN
16	81398635	C=>T	44	exonic	GAN
17	75277611	A=>C	16	UTR5	SEPT9
17	75369578	T=>C	99	UTR5	SEPT9
17	75398498	C=>T	34	exonic	SEPT9
17	75401190	G=>A	79	exonic	SEPT9
17	75494705	A=>G	58	exonic	SEPT9
17	75494746	A=>G	42	UTR3	SEPT9
17	75495065	T=>C	86	UTR3	SEPT9
17	75495397	T=>C	90	UTR3	SEPT9
17	75495523	A=>G	7	UTR3	SEPT9
18	77441442	C=>A	30	UTR5	CTDP1
18	77470834	C=>T	57	intronic	CTDP1
18	77473086	G=>A	44	exonic	CTDP1
18	77473127	C=>T	47	exonic	CTDP1
18	77474921	G=>A	39	exonic	CTDP1
18	77475455	G=>A	17	exonic	CTDP1
18	77478011	C=>T	16	exonic	CTDP1
18	77513721	T=>C	144	exonic	CTDP1
19	10265312	T=>C	125	exonic	DNMT1
19	10265593	G=>T	26	exonic	DNMT1
19	10267077	T=>C	85	exonic	DNMT1
19	10270741	TGGGGGAA=>T	9		
19	10270746	GA=>G	101	intronic	DNMT1
19	10273372	T=>C	11	exonic	DNMT1
19	10904480	C=>T	5	exonic	DNM2
19	10908143	T=>A	35	exonic	DNM2
19	10939792	T=>C	14	exonic	DNM2

19	40900492	A=>C	6	exonic	PRX
19	40900865	C=>T	129	exonic	PRX
19	40901011	G=>C	39	exonic	PRX
19	40901496	T=>C	79	exonic	PRX
19	40901604	A=>G	60	exonic	PRX
19	40901614	A=>G	77	exonic	PRX
19	40902681	C=>T	14	exonic	PRX
19	40903528	G=>A	7	exonic	PRX
19	40904602	G=>A	40	exonic	PRX
19	50339737	G=>A	5	intronic	MED25
19	50713713	C=>A	17	exonic	MYH14
19	50720940	T=>C	5	exonic	MYH14
19	50726570	G=>A	97	exonic	MYH14
19	50747533	T=>C	6	exonic	MYH14
19	50752897	G=>A	14	intronic	MYH14
19	50753870	C=>T	11	exonic	MYH14
19	50760716	C=>G	93	exonic	MYH14
19	50762418	A=>G	117	exonic	MYH14
19	50771409	A=>C	29	intronic	MYH14
19	50771432	C=>T	6	exonic	MYH14
19	50771609	G=>A	90	exonic	MYH14
19	50779469	C=>T	42	intronic	MYH14
19	50780082	C=>A	5	exonic	MYH14
19	50792684	C=>T	5	intronic	MYH14
19	50792808	T=>A	14	exonic	MYH14
19	50796905	G=>A	50	exonic	MYH14
22	38369976	A=>G	120	exonic	SOX10
22	38379774	G=>A	15	exonic	SOX10
22	50885775	A=>G	51	exonic	SBF1
22	50894987	G=>A	7	exonic	SBF1
22	50895020	C=>T	22	exonic	SBF1
22	50898026	G=>A	13	exonic	SBF1
22	50901009	C=>T	10	exonic	SBF1
22	50902751	C=>T	12	intronic	SBF1
22	50913394	CGCG=>C	30		
23	70443792	C=>T	7	exonic	GJB1
23	77298857	G=>A	67	exonic	ATP7A
